# Validation of cardiovascular magnetic resonance assessment of pericardial adipose tissue volume

**DOI:** 10.1186/1532-429X-11-15

**Published:** 2009-05-05

**Authors:** Adam J Nelson, Matthew I Worthley, Peter J Psaltis, Angelo Carbone, Benjamin K Dundon, Rae F Duncan, Cynthia Piantadosi, Dennis H Lau, Prashanthan Sanders, Gary A Wittert, Stephen G Worthley

**Affiliations:** 1 Cardiovascular Research Centre, Royal Adelaide Hospital & Disciplines of Medicine and Physiology, University of Adelaide, Adelaide, SA, Australia

## Abstract

**Background:**

Pericardial adipose tissue (PAT) has been shown to be an independent predictor of coronary artery disease. To date its assessment has been restricted to the use of surrogate echocardiographic indices such as measurement of epicardial fat thickness over the right ventricular free wall, which have limitations. Cardiovascular magnetic resonance (CMR) offers the potential to non-invasively assess total PAT, however like other imaging modalities, CMR has not yet been validated for this purpose. Thus, we sought to describe a novel technique for assessing total PAT with validation in an ovine model.

**Methods:**

11 merino sheep were studied. A standard clinical series of ventricular short axis CMR images (1.5T Siemens Sonata) were obtained during mechanical ventilation breath-holds. Beginning at the mitral annulus, consecutive end-diastolic ventricular images were used to determine the area and volume of epicardial, paracardial and pericardial adipose tissue. In addition adipose thickness was measured at the right ventricular free wall. Following euthanasia, the paracardial adipose tissue was removed from the ventricle and weighed to allow comparison with corresponding CMR measurements.

**Results:**

There was a strong correlation between CMR-derived paracardial adipose tissue volume and *ex vivo *paracardial mass (R^2 ^= 0.89, p < 0.001). In contrast, CMR measurements of corresponding RV free wall paracardial adipose thickness did not correlate with *ex vivo *paracardial mass (R^2 ^= 0.003, p = 0.878).

**Conclusion:**

In this ovine model, CMR-derived paracardial adipose tissue volume, but not the corresponding and conventional measure of paracardial adipose thickness over the RV free wall, accurately reflected paracardial adipose tissue mass. This study validates for the first time, the use of clinically utilised CMR sequences for the accurate and reproducible assessment of pericardial adiposity. Furthermore this non-invasive modality does not use ionising radiation and therefore is ideally suited for future studies of PAT and its role in cardiovascular risk prediction and disease in clinical practice.

## Background

Pericardial adipose tissue (PAT) is the layer of fat that surrounds the heart. It covers 80% of the heart and constitutes between 20 and 50% of its mass[1]. Historically, this adipose layer has largely been regarded as an inert layer that, at most, may provide some mechanical protection to the coronary arteries[2]. More recently however, the benign nature of this fatty tissue is being re-evaluated.

Pericardial adipose tissue is largely brown adipose tissue[3] and is divided into two layers. The visceral, epicardial fat layer is mainly located within the interventricular and atrioventricular grooves, with lesser amounts located around the atria and right ventricle. This visceral pericardial layer (or epicardial layer) originates embryologically from mesothelial cells that migrate from the septum transversum and hence obtains its vascular supply from the coronary arteries[4,5]. The paracardial fat (or mediastinal fat) is situated external to the parietal layer of the pericardium. This layer originates from the primitive thoracic mesenchymal cells and thus derives its blood supply from non-coronary sources such as the pericardiacophrenic branch of the internal mammary artery[5].

Although the pathophysiological significance of PAT remains uncertain, it has recently become the subject of increasing attention, with emerging evidence that it may be an independent predictor of obstructive coronary artery disease [6-8] and possibly acute coronary syndrome[6]. Therefore the characterisation of PAT by non-invasive imaging looms as a potentially valuable adjunct in evaluating cardiovascular risk. There are several candidate modalities for non-invasive imaging of pericardial fat with an ideal methodology providing robust and reproducible data with high accuracy when validated against *ex vivo *fat mass measurement. Transthoracic echocardiography has traditionally been used to measure epicardial adipose thickness[9], however variable image quality remains a significant limitation particularly in obese subjects: a patient cohort in whom quantification of adipose tissue may be especially relevant. More recently, cardiac computed tomography (CT)[8,10,11] and cardiovascular magnetic resonance (CMR)[12] have been applied with encouraging results. Although magnetic resonance imaging is recognised as the "gold standard" modality for imaging adipose tissue[13,14], CMR has only recently evolved to assess adiposity around the heart[12]. The accuracy of this methodology, however, is yet to be verified against actual measurement of *ex vivo *mass. In this paper we describe a novel method of assessing complete PAT by volume, and additionally, validate ovine paracardial adipose mass collected at necropsy with its corresponding CMR derived volume. Furthermore we show that volumetric assessment of adipose tissue is a more accurate measure of adipose tissue mass compared with surrogates such as adipose tissue thickness at the RV free wall.

## Methods

An ovine model was chosen to allow direct comparison between CMR-derived measurement of paracardial tissue volume and paracardial tissue mass at necropsy. In addition, a secondary analysis was performed to determine whether single thickness measurement of adipose tissue over the right ventricular (RV) free wall (a parameter routinely used in echocardiographic studies) reflects an accurate surrogate assessment compared to the corresponding adipose tissue mass measured at necropsy.

### Cohort

Approval was obtained from the Animal Ethics Committees of the Institute of Medical and Veterinary Services, Adelaide and the University of Adelaide, South Australia. Animal handling was carried out humanely by dedicated staff in accordance with the "Principles of Laboratory Care" formulated by the National Society for Medical Research and the "Guide for the Care and Use of Laboratory Animals" prepared by the Institute of Laboratory Research and published by the National Institutes of Health.

All animals underwent comprehensive CMR assessment immediately prior to euthanasia at which time heart specimens were harvested for histopathological analysis.

### Cardiovascular magnetic resonance protocol

Cardiovascular magnetic resonance images were acquired using a 1.5 Tesla system (Siemens, Sonata, Erlangen, Germany). Animals were anaesthetised and placed supine within the MR chamber. The animals were mechanically ventilated which allowed breath-hold sequences to be obtained. Pericardial adipose tissue was assessed using sequential ECG-gated steady state free precession short axis cine sequences acquired for assessment of ventricular function (TR/TE 52.05 ms/1.74 ms, flip angle 70°, matrix 256 × 150, FOV 380 mm with slice thickness 6 mm with no inter-slice gap). To validate a clinically relevant protocol that is already widely used in human studies, this study intentionally did not incorporate adipose-specific sequences and thus did not require additional imaging time.

### Image analysis

**Figure 1:**
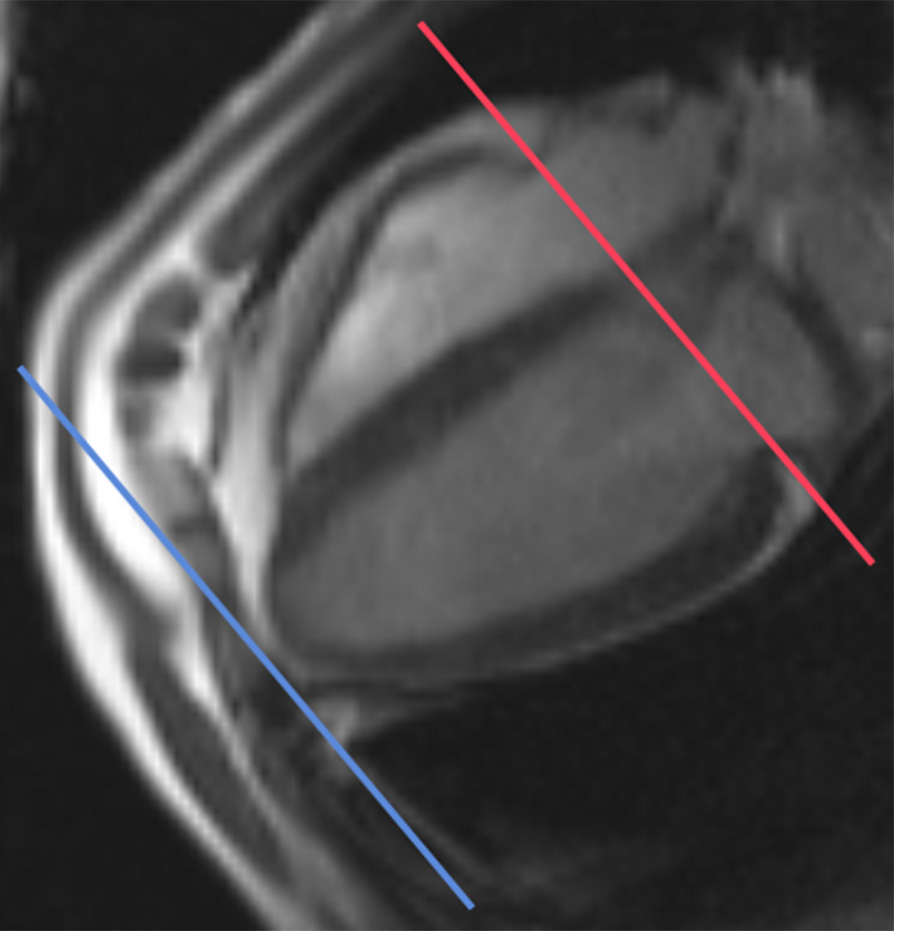
**Basal and apical slice selection landmarks**. Slice through the valve annulus was selected as basal image as this was easiest to approximate for dissection at necropsy.

**Figure 2:**
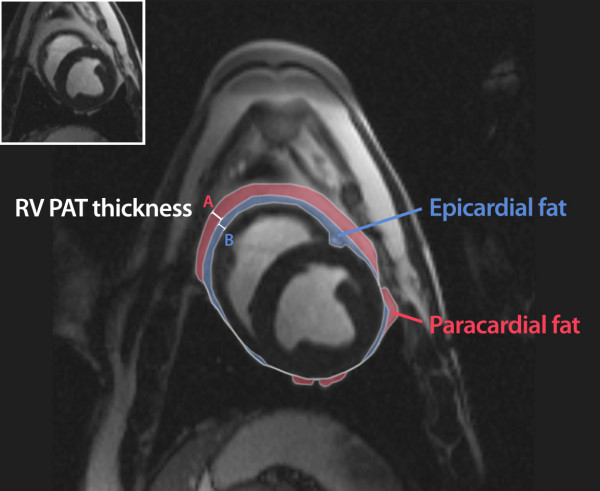
**CMR evaluation of pericardial adipose tissue**. Volumetric assessment: 3 traces, myo-epicardial border, viscero-parietal pericardial border and outer paracardial margin each subtending areas of epicardial and paracardial (and subsequently pericardial) areas which can then be converted to volumes. Thickness assessment: adipose tissue thickness measured at the midpoint of the right ventricular free wall on the mid chamber short axis image. Epicardial (A) and paracardial (B) thickness measurements depicted above.

Image analysis was carried out off-line using commercially available software, Image Pro Plus (v4 MediaCybernetics). Areas of PAT were traced on consecutive end diastolic short-axis images beginning with the most basal slice at the level of the mitral valve (Figure 1) and moving apically through the stack until the most inferior margin of adipose tissue was traced. The basal slice was taken through the mitral annulus as this was easiest point to dissect at necropsy. Three traces were made on each slice as shown in Figure 2. The first line was drawn along the myo-epicardial border. The second line was drawn at the parietal and visceral pericardial border, subtending an area of epicardial fat between this line and the myo-epicardial border. A third line was drawn along the outermost margin of paracardial fat. Epicardial, paracardial and subsequently pericardial fat areas were calculated and volumes were derived using a modified Simpson's rule.

Pericardial adipose thickness at the mid-right ventricular free wall was separately measured as per equivalent echocardiographic methodology used in previous studies evaluating pericardial adiposity[9,15]. As with volumes, adipose thickness was sequentially determined from the epicardial, paracardial and finally pericardial zones (Figure 2).

Intra- and interobserver variability was also assessed for the measures of CMR PAT thickness and volumes as described above.

### Ex vivo pericardial fat assessment

**Figure 3:**
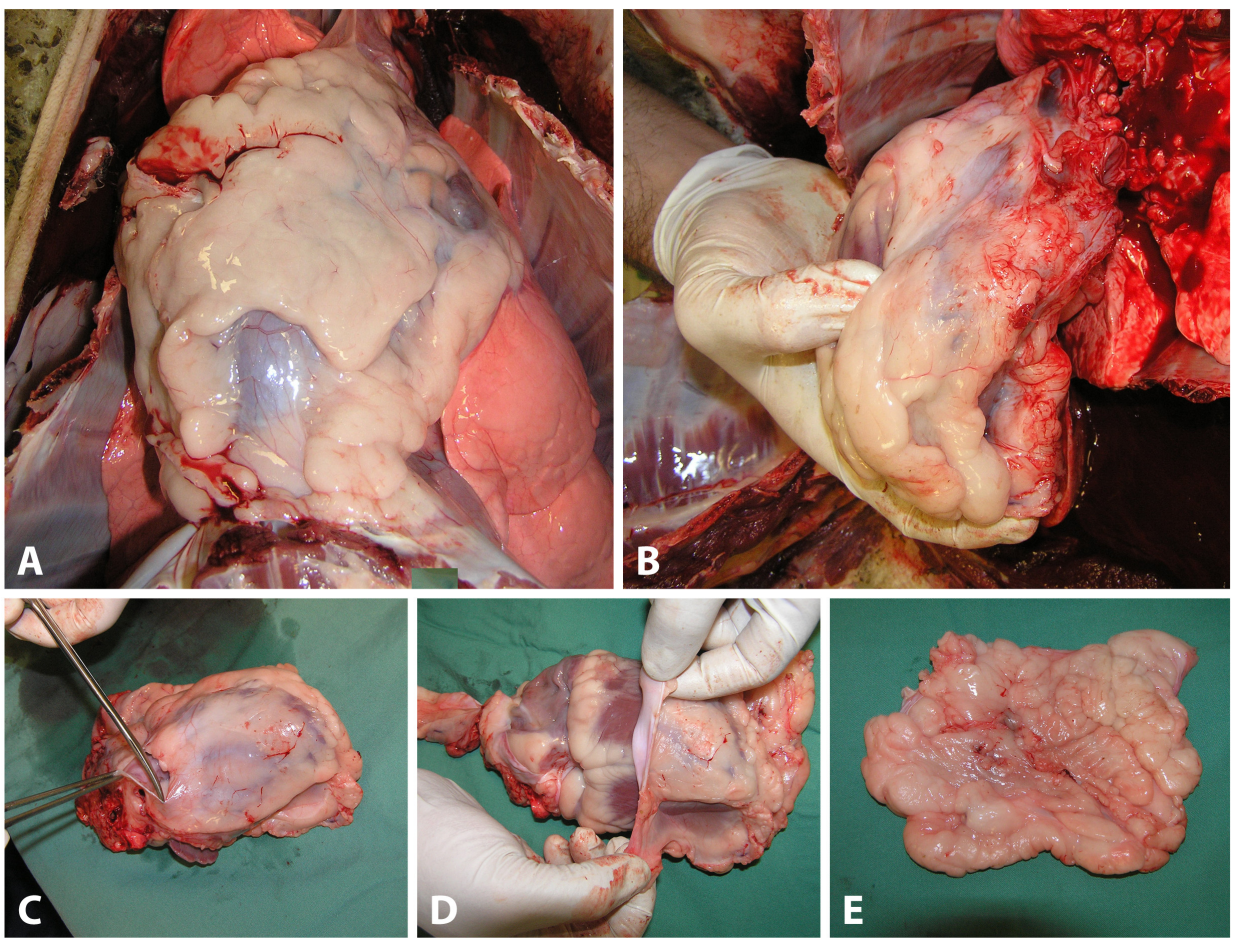
**Removal of pericardial fat at necropsy**. A) Ovine heart in situ. B) Careful removal of heart collecting adherent fat. C) Cutting through pericardium at the mitral annulus – equivalent point for CMR measurements. D) Removal of PAT from heart by peeling layers back apically. E) Complete removal of PAT before weighing.

The sheep were euthanased following their final CMR examination. The hearts were surgically excised from the thorax with particular care taken to collect all parts of paracardial fat adherent to the mediastinal cavity. As depicted in Figure 3, an incision was made circumferentially along the atrio-ventricular groove through the pericardium. Ventricular adipose tissue, not atrial adipose tissue, was assessed as this has been previously shown to be an independent predictor of coronary artery disease[8] and it is known atrial PAT is only a minor contributor to total myocardial PAT[16]. The pericardium and adherent paracardial adipose tissue was then peeled back from the heart in an apical direction and weighed immediately.

Only the paracardial fat was weighed as we found, like others have before us[17] that it is virtually impossible to accurately dissect the epicardial adipose tissue from the myocardium. Hence correlation between the epicardial component of PAT mass was not able to be undertaken with its respective CMR measurement.

### Statistical Analysis

Continuous variables are represented as means ± standard deviation or medians with interquartile ranges in brackets for data as appropriate. Statistical analyses were performed with the Statistical Package for the Social Sciences v15 (SPSS Inc, Chicago, Ill). The mass of paracardial adipose tissue at necropsy was compared with the paracardial adipose volume assessed with CMR using simple linear regression analysis. Two sided p-values of less than 0.05 were considered statistically significant. Intra- and inter-observer variability was assessed and data analysed with Bland-Altman plots, and coefficients of variation.

## Results

Eleven Merino sheep (49 ± 7 kg) were evaluated. CMR imaging was successfully performed in all animals and all images were of acceptable quality for subsequent analysis. CMR imaging acquisition time was approximately 45 minutes per animal. There were no adverse outcomes as a result of the CMR imaging process.

### Image analysis

We were able to trace, and therefore assess, the epicardial, paracardial (and therefore total pericardial adipose tissue) areas in all cases. CMR measurements of adipose tissue thickness and volumes for epicaridal, paracardial and pericardial regions are shown in table 1. The mean CMR derived PAT thickness was 0.856 ± 0.16 cm and the mean CMR derived PAT volume was 103 ± 26 cm^3^.

**Table 1:** Table of CMR and necropsy measurements of PAT. Individual CMR and necropsy measurements of epicardial, paracardial and pericardial adipose tissue in the 11 sheep studied.

	Adipose tissue thickness at mid RV			Adipose tissue volume			Pericardial adipose tissue mass
	Epicardial (mm)	Paracardial (mm)	Pericardial (mm)	Epicardial (cm^3^)	Paracardial (cm^3^)	Pericardial (cm^3^)	At necropsy (g)

1	5.21	3.52	8.73	27.493	56.957	84.45	99.22
2	4.62	4.28	8.9	59.37	64.56	123.93	141.68
3	4.45	4.74	9.19	49.194	61.122	110.316	142.4
4	5.63	3.81	9.44	52.836	90.852	143.688	221.2
5	3.39	4.84	8.23	38.21	65.614	103.824	145.3
6	6.08	3.91	9.99	56.521	83.723	140.244	228.3
7	5.28	5.16	10.44	21.476	57.436	78.912	91
8	5.39	4.02	9.41	34.18	57.806	91.986	125.1
9	4.18	2.25	6.43	24.552	60.252	84.804	125
10	0.81	4.07	4.88	30.036	78.144	108.18	139.4
11	4.97	3.28	8.25	19.569	40.719	60.288	72.18

### Ex vivo pericardial fat assessment

In all animals, *ex vivo *paracardial adipose tissue was able to be excised and weighed as described (Figure 3). Paracardial adipose tissue mass was highly variable across the eleven sheep (Table 1). The mean *ex vivo *mass of paracardial adipose tissue in this cohort was 139 ± 49 g.

### Statistical Analysis

**Figure 4:**
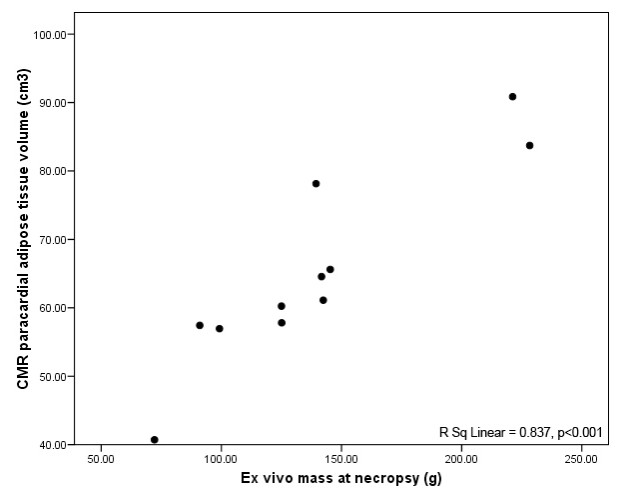
**CMR assessed PAT volume versus mass**. Linear regression of CMR assessed PAT volume (cm^3^) with *ex vivo *mass at necropsy (g). R^2 ^= 0.892, p < 0.001.

**Figure 5:**
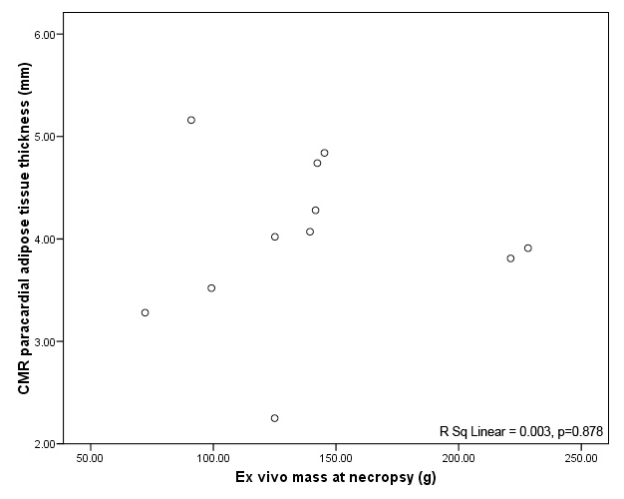
**CMR assessed PAT thickness at right ventricular free wall versus mass**. Linear regression of CMR assessed PAT thickness at right ventricular free wall (cm) with *ex vivo *mass at necropsy (g). R^2 ^= 0.334, p = 0.63.

In regards to the primary hypothesis, there was a strongly positive correlation between CMR derived paracardial adipose tissue volume and *ex vivo *paracardial adipose mass at necropsy (R^2 ^= 0.89, p < 0.001) (Figure 4). Notably, neither RV free wall paracardial nor pericardial fat thickness correlated with *ex vivo *PAT mass (R^2 ^= 0.003, p = 0.878 and R^2 ^= 0.33, p = 0.63 respectively) (Figure 5).

Intra and inter-observer coefficients of variation for CMR PAT volumes were 3.5% and 4.9% respectively (Table 2). The intra and inter-observer coefficients of variation for CMR PAT thickness were higher at 6.7 and 7.6% respectively (Table 2).

**Table 2:** Intra and interobserver reproducibility of PAT components.

		Mean (x, y)	Mean (x-y)	Coefficient of variation
		*Intra-observer variability*		
Thickness		[cm]	[cm]	[%]
	Epicardial	0.485	0.028	7.6
	Paracardial	0.398	0.015	7.3
	Pericardial	0.883	0.034	6.7
Volume		[cm^3^]	[cm^3^]	[%]
	Epicardial	38.925	0.384	4.8
	Paracardial	61.900	0.693	4.7
	Pericardial	103.174	1.077	3.5

		*Inter-observer variability*		

Thickness		[cm]	[cm]	[%]
	Epicardial	0.499	0.023	10.4
	Paracardial	0.404	0.030	8.1
	Pericardial	0.904	0.053	7.6
Volume		[cm^3^]	[cm^3^]	[%]
	Epicardial	40.206	0.972	6.3
	Paracardial	63.846	1.605	5.3
	Pericardial	100.718	2.576	4.9

Intra and interobserver reproducibility data for thickness and volume measurements of epicardial, paracardial and pericardial adipose tissue.

In assessing PAT, the reproducibility of total PAT volume was higher than for the individual epicardial and paracardial measures.

## Discussion

This is the first study to describe an accurate and reproducible CMR technique that is capable of measuring not only total PAT by volume but also its constituents, epicardial and paracardial adipose tissue. In addition, we have shown that CMR assessment of adipose tissue volume displays a strong correlation with its corresponding *ex vivo *adipose mass, validating its use as a robust measure of cardiac adipose tissue. Further, we have shown that conventional RV free wall adipose thickness measurements do not accurately quantify total PAT. Finally, we have described a method for assessing PAT which intentionally utilises image sequences that would normally be acquired during a standard CMR protocol for myocardial structure and function assessment. The images used were not dedicated adipose-specific sequences as is the case in other studies[9,12] and hence our methodology may be applied to a number of clinically indicated human scans without the need for additional imaging time.

There is growing evidence to support an important role for PAT in the pathophysiology of cardiovascular disease. Although there has recently been a resurgence of interest in this area, the initial descriptions of pericardial adiposity date back hundreds of years. Its initial association with adverse events was described by Senac in 1783 who described "a case of sudden death in which fat spread over the heart extinguishing its movement"[18]. Furthermore the diagnosis of 'adipositas cordis' was a fashionable diagnosis in Victorian times, although the continued association with sudden cardiac death was more than likely related to sudden coronary thrombotic occlusion rather than myocardial fatty infiltration[18]. It was James Herrick's seminal recognition of the clinical syndrome of myocardial infarction in the early 19^th ^century that led to diminished interest in pericardial adiposity.

Much of the initial work performed to quantify epicardial fat was performed at autopsy[1,17,19,20], with the epicardial adipose tissue weighing on average 50 grams and thus representing approximately 20% of the heart's mass. More recently, echocardiography has been used to evaluate epicardial fat by measuring its thickness over the RV free wall: a prominent site for adipose tissue around the heart[9,21]. Indeed epicardial fat thickness has been shown to be a predictor of intra-abdominal visceral fat[9,15], BMI[7,22], left ventricular mass[23] and insulin resistance[24]. Despite this, our ovine study did not find a correlation between CMR measures of RV free wall epicardial or pericardial adipose thickness and the actual mass of pericardial fat at necropsy. We acknowledge however, that there may still be some utility in using epicardial fat thickness in ongoing research of cardiovascular risk assessment, particularly given the accessibility and affordability of transthoracic echocardiograpy compared to CMR. However, the imperfect relationship between this echocardiographic index and PAT, in addition to its lower reproducibility, mean that much larger numbers of patients will be required in such studies. Our CMR technique allows future studies of PAT to show potential alterations or even changes in PAT in response to various therapeutic interventions without the need for prohibitively large sample sizes.

While echocardiography has obvious benefits it also has limitations: most notably its reliance on optimal imaging windows which are notoriously difficult in obese patients[25]. In one study, evaluating over 900 patients from an outpatient echocardiography service, 14% of obese subjects had poor echocardiographic images[26]. This is relevant given that these high-risk patients may ultimately benefit from cardiovascular risk prediction assessment from non-invasive characterisation of epicardial and/or pericardial fat. By comparison, CMR is heralded for its ability to provide excellent tissue resolution in such patients. Echocardiography is also limited in its ability to measure the complete pericardial adipose layer and in particular, to determine total pericardial volume. Volumetric assessment of PAT by CT has been shown to predict the presence of significant coronary artery disease[8] and also to be associated with left ventricular structure and function[10] in subgroups of the Framingham Heart Study. However unlike CMR, cardiac CT has the limitation of exposing patients to ionising radiation, which becomes an increasingly important consideration if patients are subjected to serial imaging.

An intriguing issue that has remained unresolved in the area of pericardial adipose assessment relates to the importance of differentiating between the epicardial and paracardial layers of adipose. The mechanistic relevance of these two zones of fat appears to be quite distinct. They have different embryological origins and vascular supply[4,5]. The epicardial fat layer expresses higher levels of pro-inflammatory mediators than subcutaneous fat in patients with established coronary artery disease suggesting a pathophysiological relationship[27,28]. Pericardial adipose tissue may also have a detrimental effect on the coronary vasculature due to mechanical restriction and impairment to diastolic coronary flow[29]. As such, the paracardial layer may have greater importance because it remains the main component of total PAT, as confirmed by the current study. Further studies are required to evaluate the relative clinical importance of the individual adipose layers.

## Limitations

Although it was necessary for this study to be performed in animals to allow for validation of *in vivo *CMR against *ex vivo *tissue assessment, we do not have reason to doubt the applicability of our findings to the human clinical scenario. Furthermore, we deliberately did not use pre-defined, fat-specific imaging sequences in our CMR protocols because it was our intention to utilise the exact images which would otherwise be acquired in a routine clinical investigation. Whilst this CMR protocol did not utilise inter-slice gaps in the short axis stack, unpublished data analysed in other studies[30] suggests it would be unlikely to have a significant effect on our results.

## Summary

A simple CMR volumetric technique using standard clinical steady state free precession imaging is the most accurate and reproducible tool currently available for the assessment of pericardial adipose tissue. Furthermore this non-invasive modality does not use ionising radiation and therefore is ideally suited for future studies of pericardial adipose tissue and its role in cardiovascular risk prediction and disease in clinical practice.

## Competing interests

The authors declare that they have no competing interests.

## Authors' contributions

SW, MW and AN designed the study. CP and RD provided input into CR sequence selection. AC, PP, DL and AN collected necropsy data. AN and AC traced the image contours. SW, MW and AN completed the statistical analysis of the data. SW, MW and AN drafted the article. SW, MW and AN created the figures and images. PP, BD, PS and GW were responsible for critical revision of the article and important intellectual content. All authors read and approved the final manuscript.
